# Negative Emotions in Migraineurs Dreams: The Increased Prevalence of Oneiric Fear and Anguish, Unrelated to Mood Disorders

**DOI:** 10.1155/2014/919627

**Published:** 2014-06-24

**Authors:** F. De Angeli, C. Lovati, L. Giani, C. Mariotti D'Alessandro, E. Raimondi, V. Scaglione, D. Castoldi, E. Capiluppi, C. Mariani

**Affiliations:** Neurological Unit, Headache Center, L. Sacco Hospital, University of Milan, Via Giovanni Battista Grassi, No. 74, 20157 Milan, Italy

## Abstract

*Background*. Migraineurs brain has shown some functional peculiarities that reflect not only in phonophobia, and photophobia, but also in mood and sleep. Dreaming is a universal mental state characterized by hallucinatory features in which imagery, emotion, motor skills, and memory are created de novo. We evaluated dream contents and associated emotions in migraineurs. *Materials and Methods*. 412 subjects: 219 controls; and 148 migraineurs (66 with aura, MA; 82 without aura, MO), and 45 tension type headache patients (TTH). A semistructured retrospective self-reported questionnaire was used to evaluate dreams. The Generalized Anxiety Disorder Questionnaire (GAD-7), and the Patient Health Questionnaire (PHQ-9) were administered to evaluate anxiety and depression. *Results*. Migraineurs showed increased levels of anxiety (*P* = 0.0002 for MA versus controls, *P* = 0.004 for MO versus controls). Fear and anguish during dreaming were more frequently reported by migraine patients compared to controls, independently by anxiety and depression scores. *Discussion*. The brain of migraineurs seems to dream with some peculiar features, all with a negative connotation, as fear and anguish. It may be due to the recorded negative sensations induced by recurrent migraine pain, but it may just reflect a peculiar attitude of the mesolimbic structures of migraineurs brain, activated in both dreaming and migraine attacks.

## 1. Background

Migraine is a very frequent primary headache, especially among women (about 15–18% of women are affected versus 6–8% of men) [1]. Many psychological and physical elements may modulate frequency and intensity of migraine attacks in migraineurs, such as sleep behavior [2], hormones, mood, and anxiety. Dreaming is a universal mental state characterized by hallucinatory features in which imagery, emotion, motor skills, and memory are created de novo. All these aspects can simulate waking experiences but they are generated by different neurobiological processes. Dream contents are widely different and a series of endogenous and exogenous factors may influence this variability, first of all mood, anxiety, well-being, and sleep quality. An adequate sleep behavior is able to influence migraine [2] mood and anxiety and vice versa. Relationship between dreaming activity and migraine is still unexplored.

## 2. Aim of Study

The aim of the paper is to study contemporary anxiety and mood in different kinds of headache and to evaluate the relationships between migraine and dream contents and related emotions, independently by anxiety and mood disorders.

## 3. Materials and Methods

We enrolled 412 subjects, of which 219 were noncephalgic controls and 193 headache outpatients consecutively evaluated in the headache center of the L. Sacco Hospital, Milan, Italy. Migraine was diagnosed in 148 headache patients while 45 had tension type headache (15 males and 30 females, mean age 37.9 ± 15.4). Migraine with aura (MA) was diagnosed in 66 subjects (16 males and 50 females; mean age 38.4 ± 15.5) and migraine without aura was found in 82 (19 males and 63 females; mean age 37.4 ± 14.6).

Clinical and anamnestic data were obtained by a digital questionnaire.

- Headache diagnosis was made according to ICHD-II criteria.
- A semistructured ad hoc questionnaire was used to evaluate dreaming activity. Data come from participant's retrospective self-reported information concerning their dream experiences. In this study we evaluated dream contents and dream related emotions as follows.
  - Dream content was assessed by asking patients to choose the five most frequent contents of their dream, from a list of possible topics:* drinking, eating, flying, falling, numbers, death or diseases, blood/cuts, love, sex, crying, fire and flames, the crowd, rain/storm/snow/wind, praying, accidents/dangerous situations, dancing, discussing, fighting, travelling, the own home, elevator, the family home, a church, a cemetery, the job/role, friends and relatives, money, the high school, a street, devil/monsters, famous people, countryside/wood/forest, unknown places, sea and seaside, mountains, and others.*
  - Emotional features associated with the oneiric activity were investigated by asking patients which emotions were more frequently felt during dream. They could choose, without limits of answers, between* serenity, anger, sadness, fear, happiness, anguish, and excitation.*
- The Generalized Anxiety Disorder Questionnaire (GAD-7) was used to study anxiety and the Patient Health Questionnaire (PHQ-9) to evaluate mood. GAD-7 and PHQ-9 are self-administered screening and diagnostic tools for mental health disorders, able to improve the recognition of depression and anxiety rate.

### 3.1. Statistical Analysis

- Chi square test with Bonferroni correction for multiple tests was used to compare dream features distribution between groups: nonheadache controls (ctrl), tension type headache (TTH), migraine with aura (MA), and migraine without aura (MO).
- Student's *t*-test was applied to compare mean age and mean PHQ-9 and GAD-7 scores between different diagnostic groups and between groups with different dream characteristics.

## 4. Results

### 4.1. Anxiety

Mean anxiety scores, evaluated by GAD-7 test, are summarized in Table 1. Comparing clinical groups with controls, MA and MO groups showed a significantly increased level of anxiety (resp., *P* = 0.0002 MA versus control, *P* = 0.004 MO versus control). No differences of anxiety level emerged between TTH patients and controls (*P* = 0.5 NS at chi square test). The same differences were observed after grouping by gender (migraineurs versus controls: *P* = 0.002 among men and *P* = 0.004 among women). A more detailed comparison of diagnostic groups among women and men was not possible considering the small subgroups dimension.

No difference in terms of median age between groups with and without anxiety was showed.

### 4.2. Mood

Mean PHQ-9 scores are reported in Table 1. A slight difference seems to emerge by the comparison between MA and MO groups with controls (resp., *P* = 0.05 and 0.01), but these differences are not confirmed after Bonferroni correction for multiple comparisons. No differences were found between TTH patients and controls (*P* = 0.6 NS).

Female migraineurs showed a depressed mood if compared with female controls (*P* = 0.03). No similar differences were found among men. A more detailed comparison of diagnostic groups among women and men was not possible considering the small subgroups dimension.

No difference in terms of median age between groups with and without depressed mood was shown.

### 4.3. Oneiric Activity: Dreamers Distribution

**Figure 1:**
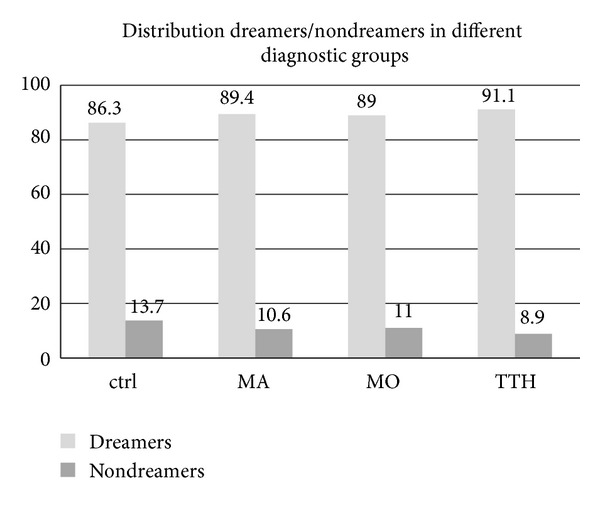


Frequency of subjects able to reevocate dreams at awakening was similar in all the groups (as reported in Figure 1.) ranging from 86 to 91%: dreamers were 189 out of 219 controls, 59 out 66 MA patients, 73 out of 82 MO subjects, and 41 out of 45 TTH patients. No differences emerged between groups in terms of distribution of dreamers/nondreamers (*χ* ^2^ = NS).

### 4.4. Oneiric Activity: Dream Contents

The only content (out of the proposed list of contents) that showed a statistically significant difference between diverse diagnostic groups is the sensation of “falling” experienced during dream. It was reported by 63 out of 219 controls (29%), 32 out of 66 MA patients (48.5%), 35 out of 82 MO patients, and 8 out of 45 TTH subjects (*P* = 0.0005 comparing the 4 groups). If considered as a single group, 45% of migraineurs (MO + MA) have falling sensation during dreams, significantly more frequently with respect to controls (29%) and TTH subjects (18%) independently by gender. No differences between TTH and ctrl were observed. No differences were observed at *t*-test comparing subjects with and without the falling sensation during dreams in terms of anxiety and depression levels. No difference in terms of median age between groups with and without falling sensation during dreams was showed.

### 4.5. Oneiric Activity: Dream-Associated Emotions

The study investigated the possible emotions associated with dreams. Serenity, anger, fear, sadness, happiness, anguish, and excitation were the possible options (multiple answers allowed).

The comparative analysis between the four groups showed a statistically significant difference (*P* = 0.005) only with regard to the sensation of fear during dreaming. This sensation was reported by 63 controls (28.8%), 27 MA patients (41%), 39 MO patients (47.6%), and 22 out of 45 TTH subjects (48.9%). All headache groups were different if compared with controls (migraineurs versus controls: *P* = 0.001 at *χ* ^2^ test; TTH versus controls: *P* = 0.004 at *χ* ^2^ test). No differences were observed at *t*-test comparing mean anxiety and depression levels between subjects with and without fear emotion during dreams. After grouping by gender no difference was observed because of a different distribution of fear sensation among male and female groups.

No difference in terms of median age between groups with and without fear emotion during dreams was showed.

A slight difference (*P* = 0.03 at *χ* ^2^ test) was found with regard to the sensation of anguish that was reported by 39.7% of controls, 51.5% of MA, 54.9% of MO, and 35.5% of TTH patients. Migraineurs showed a slightly greater proportion of patients that report sensation of anguish during dream with respect to controls (controls versus migraineurs: *P* = 0.01 at *χ* ^2^ test). There were no differences between TTH and control. Mean anxiety and depression levels were comparable between subjects with and without anguish emotion during dreams. The observed difference in terms of anguish sensation during dreams was present also among the men subgroup while it was not confirmed among women.

No difference in terms of median age between groups with and without anguish emotion during dreams was shown.

Differences observed comparing males and females groups should be examined with bigger samples and more homogenized migraine types groups.

## 5. Discussion

The larger proportion of studies concerning dreaming focused generally on psychiatric aspects of the oneiric activity with an amount of elements of nonstrictly scientific areas such as philosophy, religion, or paranormal studies. Furthermore, a scientific approach to dreams is made more complex by the lack of tools to explore the oneiric activity.

There are increasing evidences that in migraine the functional connectivity between pain-modulating circuits and the limbic system is altered [3]. The mesolimbic system, that seems to have a pivotal role in migraine, is activated also during sleep with relevant influence on memory processes, REM sleep, and dreaming, probably with the function to put in evidence information with high emotional or motivational relevance [4]. Furthermore, dysfunctional corticolimbic structures have been implicated in disorders of visceral hypersensitivity [5] such as irritable bowel syndrome and migraine [5] that share many similarities, including chronic and recurrent pain, somatic and psychiatric comorbidities, and central sensitization. This visceral hypersensitivity, which is present in migraine and in several other clinical conditions, is regulated by central neural mechanisms that are incompletely understood and influenced by stress and anxiety (initiating or exacerbating factors).

On these theoretical bases we hoped that the study of dreams among migraineurs might show new peculiar aspects of the migraineurs brain that is known to be a particular hyperexcitable and hypometabolic brain. Effectively, some peculiar elements emerged. The first observation was that the percentage of subjects able to recall dreams at awakening was similar in headache patients and in controls, even if migraineurs may complain of an increased amount of nocturnal awakenings and a delayed sleep onset, as shown by a previous study [2]. On the other hand, qualitative diversities in terms of contents and dream-associated emotions characterizing migraine patients emerged from our analyses. In fact, migraineurs seem to have, during dreams, an increased amount of negative sensations, like fear and anguish, and contents, such as the perception to fall. These elements suggest that in the brain of migraineurs some cerebral structures, such as prefrontal cortex, mesolimbic areas, amygdala, and hypothalamus, involved in both dream and migraine biology, may have some peculiar characteristic, although it is not possible to infer if they are part of the dysfunctional state of migraine or are caused by migraine transformation. The particular frequency of negative contents and sensations during dreams in migraineurs, independently from anxiety and mood disorders, may also suggest that the recurrent perception of pain may be recorded in memory and recalled as negative emotions and sensations during oneiric activity.

Our results may offer a new point of view on the peculiarity of migraine brain as far as the relevance of these archaic brain structures in its biology.

## Figures and Tables

**Table 1:** GAD-7 and PHQ-9 average scores in different diagnostic groups.

	Anxiety	Depression
Control	9.3	6.2
MA	12.4	7.8
MO	11.3	7.3
TTH	9.6	5.9
